# Epidemiologic Observations from Passive and Targeted Surveillance during the First Wave of the 2009 H1N1 Influenza Pandemic in Milwaukee, WI

**DOI:** 10.3390/v2040782

**Published:** 2010-03-25

**Authors:** Swati Kumar, Michael J. Chusid, Rodney E. Willoughby, Peter L. Havens, Sue C. Kehl, Nathan A. Ledeboer, Pippa Simpson, Meredith VanDyke, Elizabeth Davis, Kate Gaffney, Shun-Hwa Li, Michael E. Bose, Kelly J. Henrickson

**Affiliations:** 1 Midwest Respiratory Virus Program (MRVP), Suite C450, Pediatric Infectious Diseases, Children’s Hospital of Wisconsin, P.O. Box 1997, Milwaukee, WI 53201-1997, USA; E-Mails: mvandyke@mcw.edu (M.V.); edavis@mcw.edu (E.D.); kgaffney@mcw.edu (K.G.); mbose@mcw.edu (M.E.B.); khenrick@mcw.edu (K.J.H.); 2 Department of Pediatrics, 8701 Watertown Plank Road, Milwaukee, WI 53226, USA; E-Mails: mchusid@mcw.edu (M.J.C.); rewillou@mcw.edu (R.E.W.); plhavens@mcw.edu (P.L.H.); psimpson@mcw.edu (P.S.); sli@mcw.edu (S.-H.L.); kskehl@mcw.edu (S.C.K.); 3 Department of Pathology, Medical College of Wisconsin, Wisconsin, USA; E-Mail: nledeboe@mcw.edu (N.A.L.); 4 Childrens Research Institute, 8701 Watertown Plank Road, Milwaukee, WI 53226, USA; 5 Childrens Hospital of Wisconsin, 8701 Watertown Plank Road, Milwaukee, WI 53226, USA; 6 Dynacare Laboratories, 8701 Watertown Plank Road, Milwaukee, WI 53226, USA

**Keywords:** influenza, pandemic, H1N1

## Abstract

The first wave of the 2009 influenza H1N1 pandemic (H1N1pdm) in Milwaukee, WI has been recognized as the largest reported regional outbreak in the United States. The epidemiologic and clinical characteristics of this large first wave outbreak from April 28^th^ 2009–July 25^th^ 2009, studied using both passive and targeted surveillance methodologies are presented. A total of 2791 individuals with H1N1pdm infection were identified; 60 % were 5–18 years old. The 5–18 year and 0–4 year age groups had high infection (1131 and 1101 per 100,000) and hospitalization (49 and 12 per 100,000) rates respectively. Non-Hispanic blacks and Hispanics had the highest hospitalization and infection rates. In targeted surveillance, infected patients had fever (78%), cough (80%), sore throat (38%), and vomiting or diarrhea (8%). The “influenza like illness” definition captured only 68 % of infected patients. Modeling estimates that 10.3 % of Milwaukee population was infected in the first wave and 59% were asymptomatic. The distinct epidemiologic profile of H1N1pdm infections observed in the study has direct implications for predicting the burden of infection and hospitalization in the next waves of H1N1pdm. Careful consideration of demographic predictors of infection and hospitalization with H1N1pdm will be important for effective preparedness for subsequent influenza seasons.

## 1. Introduction

On April 21, 2009, the Centers for Disease Control (CDC) reported emergence of a novel influenza A (H1N1pdm) virus in the United States [1]. Institution of typing and subtyping of all influenza A positive specimens at the Medical College of Wisconsin [Midwest Respiratory Virus Program (MRVP)] and the two affiliated academic hospitals, Children’s Hospital of Wisconsin (CHW) and Froedtert Hospital [Dynacare Laboratories (DL)] within 3 days of the first cluster of case reports [2] identified the presence of the virus in Milwaukee on April 28, 2009. This paper expands an early report [3] of the subsequent outbreak, and includes targeted surveillance data. The first wave of the 2009 influenza H1N1 pandemic (H1N1pdm) in Milwaukee, WI has since been recognized as the largest reported in the United States.

As of July 24^th^, a total of 43,771 confirmed or probable cases of H1N1pdm infection in the United States were reported by the CDC, of which 6222 were in Wisconsin [4]. Milwaukee County (MC) accounted for the highest number of cases (N = 4029) in WI [5]. The epidemiology of H1N1pdm infections in in-patient and out-patient MC populations was studied using both targeted and passive influenza surveillance programs. Infection and hospitalization rates and demographic characteristics of the outbreak during weeks 17–29 (April 26^th^ 2009– July 25^th^ 2009) are presented. Data from the Milwaukee outbreak were analyzed to predict the burden of infection in MC populations, by age in the next wave of the pandemic.

## 2. Results

### 2.1. Passive Surveillance

A total of 9176 patients were tested for influenza from 26^th^ April 2009 to July 25^th^, of which 2755 (30%) were positive for influenza A. Of these 2755 influenza A positive specimens, 2741 were H1N1pdm and 14 were seasonal subtypes. There were 2238/6138 (36.4%) influenza A positive children and adolescents (< 19 years) compared to 517/3038 (17%) positive adults (≥ 19 years). Of the 2238 children and adolescents with influenza A infections, 2229 (99.6%) were identified as confirmed or probable novel H1N1 viruses, 9 (0.4%) were seasonal H1N1, none were H3N2. Of the 517 adults positive for influenza A, 512 (99%) were identified as confirmed or probable novel H1N1, 3 were seasonal H1N1 (0.6%) and 2 were seasonal H3N2 (0.4%).

The ages of H1N1pdm positive subjects ranged from two weeks to 77 years (median, 9.7 years). Males comprised 50.1% of the 2693 H1N1pdm positive cases whose gender was known. Of all males tested for influenza (N = 4123), 33% were positive and of all females tested for influenza (N = 5005), 27% were positive (p < 0.001).

**Figure 1.**
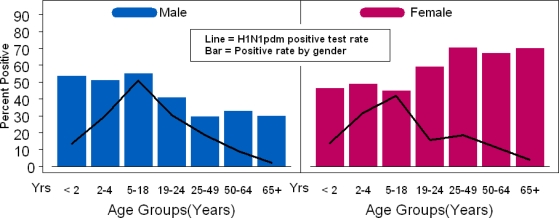
Age distribution of patients infected with influenza A H1N1pdm. Lines indicate H1N1pdm positive rate (e.g., number of patients positive/number of patients tested) by age group. Bars indicate the proportion of all patients positive in a given age group by gender (e.g., males plus females = 100%).

The H1N1pdm positive test rate by age and gender was calculated (Table S-1). Children of ages 5–18 years had the highest positive test rate, which did not differ by gender. However, for the age groups 19–24 years, 25–49 years, 50–64 years and ≥65 years, females accounted for higher proportions of positives (4.6%, 16.7%, 3.6% and 0.5%) respectively than males (3.2 %, 6.9%, 1.7% and 0.2%)[p<0.001 in the 19–24 year old age group] (Figure 1).

**Figure 2.**
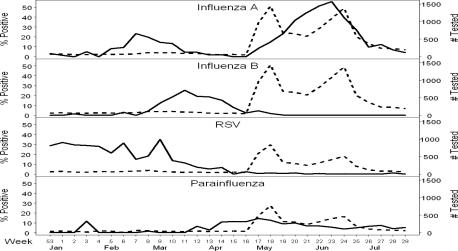
Respiratory virus activity over the 2008–2009 winter, spring and summer seasons by week in Milwaukee, Wisconsin. The total number of samples tested (depicted using dashed line) and percent positive (depicted using the solid line) are depicted for four viruses, detected using multiplex molecular assays.

The distribution of influenza A subtypes during the pre-pandemic and pandemic surveillance periods (weeks 3–29) was examined (supplementary appendix Figure S-1). Human seasonal subtypes were not identified after week 22. The contemporaneous prevalence of influenza B, respiratory syncytial virus (RSV) and parainfluenza viruses in the community is displayed in Figure 2. H1N1pdm virus remained the most prevalent of the assayed viruses, with weekly sample detection rate reaching a peak of 55% (N tested = 1087) during week 23. Parainfluenza viruses were the major co-incident seasonal viruses circulating with a peak of 14.4% detection in week 17 and 3.7%–7.4% detection during weeks 21–29 of the study. RSV and influenza B remained sporadically detectable at very low levels with highest detection of 1.3% and 0.2% respectively during weeks 21–29. Adenovirus was detected at rates up to 7.5% (N tested = 225); other respiratory viruses have not been studied.

**Figure 3.**
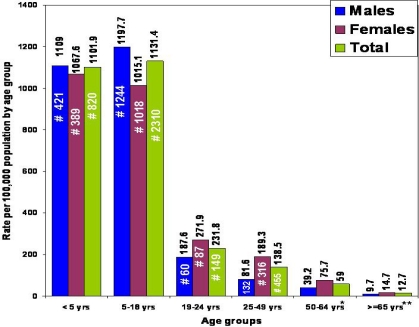
H1N1pdm Infection Rate per 100,000 Population, by age group in Milwaukee County, WI, from April 26, 2009 to July 25, 2009. Rate per 100,000 shown as numbers above bars; #, number of cases; * N (50–64 year age group) = 32, 67, 101 for Males, Females and Total (including gender not known). ** N for ≥ 65 years = 4, 10, 14 for Males, Females and Total (including gender not known).

The rates of H1N1pdm infection per 100,000 people by age and gender, estimated using the number of cases of H1N1pdm identified through passive surveillance in MC (and extrapolated to reflect true numbers for MC, [5]) are shown in Figure 3 and Table S-2 (supplementary appendix). Denominators used were derived from 2008 Census Bureau population estimates for MC [14]. Males had a higher estimated rate of H1N1pdm infection than females in age groups <5 and 5–18 years while females had higher rates for all other age groups, being highest at 2.3-times the rate in males for the 25–49 year old age group. Of the 2741 infected individuals, 48 were excluded from this analysis due to missing gender.

**Figure 4.**
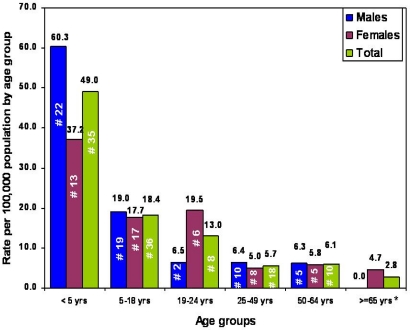
H1N1pdm associated Hospitalization Rate per 100,000 Population by age group in Milwaukee County, WI, from April 26 2009 to July 25 2009; Rate per 100,000 shown as numbers above bars; #, number of cases; * N for ≥ 65 years = 0, 3, 3 for Males, Females and Total (including gender not known).

Rates of H1N1pdm influenza associated hospitalization by age group, gender, and race are shown in Figure 4 and Table S-3 (supplementary appendix). During the period of the study, 121 H1N1pdm associated hospitalizations occurred at 2 large hospitals serving MC. Of these, 82 were <19 years of age. Eleven of the 121 hospitalized patients who were not residents of MC were excluded from further analysis. Significant differences in hospitalization rates were observed between genders in the 19–24 year old and ≥65 year old age groups (rate in females three-times and rate in males 4.7-times). Because of lack of race data in adults, hospitalization rates by race were only possible for pediatric cases. The highest rates per 100,000 were in non-Hispanic blacks (18.9) followed by Hispanics (14.9) and lowest in non-Hispanic whites (2.5).

A total of five deaths occurred, of which two were in 5–18 year olds and one each in the 25–49 year, 50–64 year and ≥ 65 year age groups. Four of the five deaths were females.

Analysis of weekly detection rates over time showed that the 5–18 year age group made up 48%–72% of all cases during the first nine weeks of the pandemic, and 30%–45% of cases in the last four weeks reported (Figures S-2, S-3 in supplementary appendix). Positive detection rates declined from 16.5%–71.4% (peak of 73%) in the first eight weeks to 9.7%–27.5% during the last four weeks (supplementary appendix, Figure S-4), p=<0.01 for all age groups except ≥65 (p=0.99). Population based infection rates by week showed the peak and subsequent decline in infection rate per 100,000 in 5–18 year old children to precede those in all other age groups (supplementary appendix, Figure S-5).

### 2.2. Targeted Surveillance

A total of 614 children and adults were enrolled in targeted prospective surveillance, 50 (8.8%) were positive for H1N1pdm. Of the 251 (40.9%) symptomatic subjects 45 (17.9%) were positive for H1N1pdm while the 363 (59.1%) asymptomatic subjects had five (1.3 %) positive subjects. There were 121 (19.7%) who had ILI. Females had a 6.4% (N = 25) positive rate while the rate in males was 11.3 % (N = 25). Females accounted for 66% of infected cases ≥19 years and 75% of the infected asymptomatic subjects. Of infected symptomatic and asymptomatic subjects, 62% and 60% respectively were 5–18 year old, identical to results from passive surveillance. No subjects ≥50 were H1N1pdm positive (N tested = 40). Analysis of symptomatic subjects by race (available for all ages) revealed 28% of Latinos, 17% of blacks and 4% of Caucasians tested positive.

The relative prevalence of symptoms in the infected, uninfected, symptomatic, and asymptomatic populations enrolled in targeted surveillance is shown in Table 1. Cough and rhinorrhea were the most common symptoms (∼80%) followed by fever (78%). Ninety percent of infected subjects met the study criteria of being symptomatic while being “asymptomatic” conferred poor specificity (64%). In subjects with ILI, sensitivity was 68% while specificity was 84.6%. Forty eight percent of symptomatic subjects met the criteria for ILI. Four of 45 H1N1pdm positives had vomiting and/or diarrhea, but all four had concurrent fever and respiratory symptoms. None of the five infected yet asymptomatic patients had gastrointestinal symptoms; three had one respiratory symptom alone (2 had runny nose, 1 had cough which lasted for one day) and two had no symptoms at all. All symptomatic H1N1pdm patients had full resolution of their symptoms in 3–5 days without further health care utilization.

### 2.3. Predicting the Burden of Pandemic Influenza Infection in the Second Wave

Rates of ILI in Milwaukee ranged from 0.2 to 5% per week during the H1N1pdm wave. The percentage of symptomatic subjects in targeted surveillance who had ILI ranged from 25% to 65%. The weekly percentage of positives in symptomatic individuals ranged from 5% to 45% except one week (week 28) when 0/8 patients enrolled were positive. The weekly percent positives in asymptomatic subjects were 2.6%, 3.4% and 4.7% (N tested =39, 59 and 43) during the peak of the wave (weeks 21, 22 and 23 respectively) and 0% in the remaining weeks. Based on these figures and after adjustment of ethnicities sampled to the census population of MC, the total number of infected symptomatic subjects was estimated to be 40,679 and the total number of asymptomatic infected subjects was estimated to be 57,807 (Table S-4, S-5 in supplementary appendix). Overall 10.3% (all ages), and 31%, 28.3%, 4.9%, 3.5%, 1.5% and 0.4% of MC populations in age groups <5, 5–18, 19–24, 25–49, 50–64 and >65 years respectively were estimated to be infected in the first wave of the pandemic (**Table S-1**, supplementary appendix).

## 3. Discussion

We report detailed epidemiology of the first wave of the 2009 H1N1 pandemic from the largest regional outbreak in the U.S. Expansion of preexisting laboratory capabilities in the MVRP and close integration of workflow with large regional clinical laboratories allowed subtype testing and reporting in real time of up to 1500 influenza A specimens per week. The proportion of positive cases identified at the MRVP peaked at 55.6% of tested specimens in week 23 (June 7–June 13th), congruent with rates of 56.4% and 55.9% at the Milwaukee Health Department Laboratory and for the WSLH [15]. Positive cases detected and reported/week declined subsequently.

The high burden in school age children (60%) is consistent with previously described transmission dynamics of influenza [16]. Closure of schools around June 15 was followed by a 75% reduction in number of weekly cases. (Figure S-2 to S-5, supplementary appendix). Females accounted for more than 50% of cases in the ≥19 age groups even though laboratory detection rates of H1N1pdm were lower or the same in females *versus* males in these groups. This was most likely due to significantly larger numbers of females being tested (1.3–2.9 times) in the ≥19 groups. This apparent increased disease burden in ≥19 year old females likely reflects higher utilization of health care or higher levels of physician testing (e.g., of pregnant women or women caring for young infants).

Infection rates per 100,000 MC population by age were compared with national rates reported by the CDC [17] and with WI state rates [15]. Attack rates using geographic population specific denominators in our study of MC were 10 to 48 times higher than the national rates and 2–5-times higher than the rates for the rest of WI, with rates being highest in the 5–18 year old children followed closely by 0–4 year old children. The 19–24 year old age group had one-fifth the rates of the 5–18 year group and thus, accounted for only a small amount of the disease in the 5–24 year old age group [17]. This may suggest that the 19–24 year group may not need to be included in the highest priority for influenza vaccination. However, the age specific H1N1pdm positive rate in males 19–24 remained high while the rate in females dropped to the same rate as in 25–49 year olds (Table S-1 in supplementary appendix) suggesting that 19–24 year old males may be at higher risk of infection.

The hospitalization rate data for children in our study are likely to be highly accurate, given that CHW accounts for ∼96% of children hospitalized in MC. However, our adult hospitalization rates probably underestimate the true rates given that Froedtert Hospital represents only 12% of adult hospitalizations in MC. Rates using population specific denominators were 10.8 times higher for MC residents than national rates in the 0–4 year age group, 2.1 times higher for the 5–18 age group and even with underestimation for adults, 5 times higher for the 25–49 year and 50–64 year olds and 1.6 times higher for the 65 and older age groups. While numerically higher, hospitalization rates were consistent with those reported nationally. Analyses by gender showed males to have a 1.6 times higher hospitalization rates than females under 5, approximately equal rates in the 5–18 years and three-times lower hospitalization rates than females in the 19–24 year old age group. Differential rates in the 19–24 year old males and females remained unrecognized when analyzing the 5–24 year olds collectively as a single group. Fatalities also concentrated in females in this small sample.

Non-Hispanic blacks (NHB) and Hispanics had higher hospitalization rates in MC than non-Hispanic whites (NHW), trends similar to hospitalization data in Chicago during the same period [18]. Both NHB and Hispanics in MC have a higher proportion of their populations in the <5 age group at 10% and 12%, than NHW (5%). Similar age distribution dynamics exist for 5–18 year olds in MC (NHB 28%, Hispanic 27%, NHW 15%). These differences in age distributions among racial/ethnic groups in MC may have contributed in part, not only to the disproportionately higher hospitalization rates in Hispanics and NHB but also the higher infection rates in Milwaukee compared to overall rates for Wisconsin and nationally. Whether true differences in susceptibility to H1N1pdm infection or more severe illness, exist among racial/ethnic populations remains unknown at this time, but a higher prevalence of race associated underlying conditions such as hemoglobinopathies or asthma [19] in NHB may have played a role in the higher hospitalization rates observed, along with the noted age distributions. Socioeconomic status, as a proxy indicator for crowding and micronutrient status, was not captured by our surveillance.

H1N1pdm influenza associated hospitalizations per 100,000 MC population during April 26, 2009 to July 25, 2009 (weeks 17–29) were compared with influenza hospitalization rates during the 2008–2009 winter influenza season (October 1, 2008–March 28, 2009) and preceding influenza seasons reported by the Emerging Infections Program (EIP) surveillance program [20,21]. H1N1pdm was associated with higher hospitalization rates for all age groups except those ≥65 years of age. Thus, H1N1pdm hospitalization rates per 100,000 over just three months were significantly higher than reported rates for seasonal influenza over 6 months (1.7-times, 3-times, 2.3-times, 1.5-times) for age groups 0–4, 5–18, 19–49, 50–64 respectively) whereas the seasonal influenza rates were 3.5-times higher than H1N1pdm rates for ≥ 65 years. Trends for 0–4 years and 5–18 years were further confirmed by comparison with data published for hospitalizations during six previous influenza seasons nationally. Pandemic influenza associated hospitalization rates exceeded seasonal influenza associated hospitalization rates reported in 2007–2008 (40.3 and 5.5 per 100,000), which were the highest of the previous six influenza seasons (2003–2009) for the two age groups respectively.

Analyses of symptomatic and asymptomatic populations enrolled during targeted surveillance demonstrated several important clinical and epidemiologic features of H1N1pdm infection. Only 68% of infected subjects met the CDC definition of an ILI, even though fever was only a subjective requirement, whereas 90% of the infected subjects met the study definition for being “symptomatic”, underscoring the need to use more liberal criteria than ILI for measuring burden and transmission of H1N1pdm in the community. Second, inclusion of gastrointestinal symptoms in the criteria for being symptomatic did not increase sensitivity for capturing infected cases. Third, although only 1.3% of subjects in the asymptomatic group were infected compared with 17.9% of symptomatic subjects, asymptomatic subjects accounted for a significant proportion (59%) of the overall burden of pandemic H1N1 infection in the community, (Table S-4, S-5 in supplementary appendix). This group is a critically important but unrecognized reservoir that may serve to spread infection throughout the community. While the actual number of subjects in the targeted surveillance was small, surveillance emphasized asymptomatic visitors (visits for non-febrile indications, family members of an index patient) and was active during the tail of the spring H1N1pdm wave, so the accuracy (n=363) of our estimates of “asymptomatic” shedding is good. Previous studies on seasonal influenza have estimated as many as 1 in 3 infected people to be asymptomatic [22]. Studies of viral shedding in experimentally infected asymptomatic subjects have shown viral shedding to occur at lower titers than symptomatic patients [23,24]. Transmission of influenza by asymptomatic and presymptomatic subjects has not been extensively studied but there is a study implicating presymptomatic transmission of infection [25].

Effective public health planning for influenza seasons requires definition of populations at highest risk for infection and severe disease or mortality from H1N1pdm. Data from the Milwaukee outbreak demonstrated that at the peak of the outbreak nearly one in every two children tested in the 5–18 year old age group were H1N1pdm positive. Predictions for estimated infection burden in different age groups (Table S-1, supplementary appendix) in the coming influenza season suggest that ∼70% of children ≤ 18 and ≥ 95% of those 19–49 years of age could be susceptible to the second wave of H1N1pdm. We estimate that 10.3% of MC total population was infected in the first pandemic wave and significant numbers of susceptible remain. Targeting children and adolescents in the H1N1pdm influenza vaccination programs will continue to be a critical tool for mitigation of overall morbidity and mortality from pandemic and seasonal strains of influenza virus, but may be less effective if 30% of the study population might already be immune.

Although more than 98% of ≥50-year old people are estimated to be susceptible to H1N1pdm, the low infection rates observed during the first wave reaffirm preliminary data demonstrating preexisting immunity [26] and predict relative sparing of this age group in the second and subsequent waves.

There are several limitations in the study. These include the small size of the targeted surveillance population studied and possible biases due to only six surveillance sites. The small number of the infected asymptomatic subjects lead to very large confidence intervals. Additionally, for the purpose of estimating the infected and susceptible populations it was assumed that the rates of ILI are similar in different racial and demographic groups and that the subjects infected in this outbreak will be spared in the next wave.

## 4. Methods

*Passive surveillance* for H1N1pdm infections was initiated on April 27, 2009. Specimens referred to the MRVP from the CHW and DL were obtained from both in- patient and out-patient subjects of all ages from the MC region. The majority of specimens were from out-patients. In-patient specimens were collected from patients hospitalized at CHW and two adult hospitals in Milwaukee, WI. Milwaukee is the 19^th^ largest city in the United States and MC had a population of 953,328 people in 2008.

In the first two weeks of the outbreak, public health agencies recommended testing all patients with influenza like illness (ILI), but on May 11, this changed to testing only hospitalized or high-risk patients [3]. ILI was defined as fever (temperature of 100 °F [37.8 °C] or greater) and a cough and/or a sore throat in the absence of a known cause other than influenza [6]. Patients considered to be at high risk for severe H1N1pdm were the same age and risk groups as those at higher risk for seasonal influenza complications [7]. In addition, prospective patient demographic data and data for other respiratory viruses circulating in MC during the period of the outbreak were collected and analyzed. For patients with duplicate samples only the earliest dated sample is included in this analysis.

*Targeted surveillance* was initiated on May 21 2009. Subjects of all ages were approached in three community-based clinics, two emergency rooms (ER) (CHW, Froedtert Hospital) and 1 urgent care center, all located in MC. The clinics and urgent care center served socio-economic and culturally diverse populations. The CHW emergency room is responsible for ∼91% of pediatric ER visits in the County and the Froedtert ER provides 12% of adult MC ER visits. Subjects approached included those with and without respiratory symptoms and did not need to be clinic or ER patients. Subjects were considered “symptomatic” if they met any two of the following criteria: fever, cough, sore throat, coryza, or myalgia. People who had no symptoms, only one of the above symptoms, or other symptoms were considered asymptomatic for an ILI, and are referred to as “asymptomatic” in the study. Individuals who had subjective fever with cough or sore throat were considered to have an ILI and thus are a subset of those “symptomatic”. Nasal swabs were collected from study participants after obtaining informed consent. Samples were placed in M4 or M6 viral transport medium (Remel, Lenexa, KS). Specimens were transported to the MRVP laboratories (cold packs) for testing for H1N1pdm virus. All samples were processed as previously described [8]. Patients were subsequently contacted by telephone to determine duration of symptoms and whether they had required hospitalization or further medical care for their illness.

*Laboratory testing* CHW and DL performed influenza testing using multiplex real-time reverse transcription polymerase chain reaction (RT-PCR) assays for detection of influenza A, influenza B and respiratory syncytial virus (RSV) which did not subtype influenza A [9]. However, testing performed at DL was able to indicate which influenza A positive samples had abnormal test results (abnormal real time melt profiles) that subsequently correlated 100% with molecular diagnosis of H1N1pdm. Influenza A positives from DL and CHW were sent to the MRVP for specific subtype determination. The MRVP utilized previously described RT-PCR assays [10, 11] validated for detection of the H1N1pdm by the WI State Laboratory of Hygiene (WSLH) using CDC diagnostic kits [3]. A laboratory confirmed case of H1N1pdm infection was defined as anyone with a respiratory specimen positive for H1N1pdm using molecular assays validated for detecting the virus.

All samples referred to the MRVP were subtyped until June 1 2009. At that point, all influenza A specimens from CHW and DL had been confirmed as H1N1pdm for several weeks. Thereafter, only samples from targeted surveillance and hospitalized patients were subtyped, and all other influenza A positive samples were considered probable H1N1pdm. Approximately 90 of the H1N1pdm viruses detected during the first five weeks of the outbreak had whole genome sequencing performed [12] All data in the study were collected under a protocol approved by the Institutional Review Board of Children’s Hospital of Wisconsin and the Medical College of Wisconsin.

*Modeling the burden of H1N1pdm influenza infections in the 2009–2010 influenza season*. We obtained estimates of the prevalence of ILI (CDC definition) in MC from weekly surveillance reports published by the Department of Health Services, Division of Public Health, Wisconsin [13]. Using these and targeted surveillance data collected by the MRVP, we calculated (successively) the percentage of our “symptomatic” subjects who met the criteria for ILI each week, the percentage of “symptomatic” subjects in MC and number of “symptomatic” subjects in MC by week. The relative distribution of symptomatic individuals by age every week, was estimated by analyzing data (age specific distribution percents) collected from the 9176 patients enrolled in passive surveillance. The number of asymptomatic persons by age was derived by subtracting symptomatic individuals from the total respective populations. The positive rate (number H1N1pdm positive/number tested) by week determined in targeted surveillance in symptomatic subjects and asymptomatic subjects (separately) was applied to the number of subjects in the respective populations to calculate the number of infected symptomatic and asymptomatic individuals by age. We calculated and applied adjusted positive rates, weighted to allow for the differences in demographic makeup of our sample relative to the entire county population (Table S-4 to S7, supplementary appendix). Infected population numbers were then subtracted from the total MC populations to estimate the number of people by age susceptible to infection. Positive test rates observed in the passive surveillance were applied to susceptible subjects to predict the potential burden of infection by age in a subsequent wave of H1N1pdm.

## 5. Conclusions

Early detection of the pandemic virus and rapid ascertainment of relative proportions of circulating subtypes and their resistance patterns during the 2009–2010 influenza season will be critical to guiding therapeutic decisions. Timely detection will require routine use of molecular assays for rapid subtyping of influenza A viruses. Significant changes in influenza diagnostic strategies may be necessary for accurate detection of this virus by laboratories around the nation and worldwide. Given significant variation in population dynamics regionally [14], different regions in the country would be expected to be affected differentially. Careful consideration of data emerging from epidemiologic investigations will be important to formulate the most effective public health strategies for prevention and control of this infection in the ongoing and subsequent influenza seasons.

## Supplementary Materials



## Figures and Tables

**Table 1.** Prevalence of Symptoms in H1N1pdm Infected and Uninfected Subjects and “Symptomatic” and “Asymptomatic” Subjects in Targeted Surveillance.

**Symptom/s H1N**	**1pdm Positive (%) N=50**	**H1N1pdm Negative (%) N=564**	**p-value**	**“Symptomatic” (%) N=251**	**“Asymptomatic” (%) N=363**	**p-value**
Rhinorrhea	82	31.2	<0.001	75.3	7.7	<0.001
Cough	80	32.8	<0.001	82.1	5.2	<0.001
Fever	78	19.3	<0.001	59	0	<0.001
Sore throat	38	16.8	<0.001	40.2	3.6	<0.001
Headache	34	15.6	0.018	28.3	9.4	<0.001
Diarrhea	8	8.2	0.99	15.5	3	<0.001
Vomiting	8	10.5	0.99	17.1	5.2	<0.001
Nausea	8	10.3	0.99	28.3	9.4	<0.001
Myalgias	6	2	0.58	4.8	0.6	<0.001
Seizure	0	0.2	0.99	0	0.3	0.99
Rash	0	3.4	0.99	4.8	1.9	0.11
Fever and cough (no sore throat)	36	7.4	-	23.9	0	-
Fever and sore throat (no cough)	2	1.2	-	3.2	0	-
ILI**	68	15.4	<0.001	48.2	0	<0.001
No ILI	32	84.6	-	51.8	100	-
“Symptomatic”	90	36.5	<0.001	-	-	-
“Asymptomatic”	10	63.5	-	-	-	-

* “Symptomatic” subjects were those who met any two of the following criteria: fever, cough, sore throat, coryza, or myalgia

** ILI was defined as presence of subjective fever with cough or sore throat.
