# Supplementary material for: Epidemiologic Observations from Passive and Targeted Surveillance during the First Wave of the 2009 H1N1 Influenza Pandemic in Milwaukee, WI
Source: Viruses. 2010 Mar 25;2(4):782–95. doi: 10.3390/v2040782 (PMC2905828; doi:10.3390/v2040782)
Supplement: Supplementary file 1 [file viruses-02-00782-s001.pdf]

Supplementary Tables and Figures for article "Epidemiologic Observations from Passive and Targeted Surveillance during the First Wave of the 2009 H1N1 Influenza Pandemic in Milwaukee, WI", published 25 March 2010.

Table S 1. Rate of H1N1pdm Infection in Individuals Tested in Milwaukee County by Age and Gender.

**MALES***	**< 2Yrs**	**2-4 yrs**	**5-18 yrs**	**19-24 yrs**	**25-49yrs**	**50-64yrs**	**≥ 65yrs**	**Total**
Number positive	110	190	886	43	94	23	3	1349
Number tested	813	642	1702	109	474	241	142	4123
Positivity rate (%)	13.5	29.6	52.1	39	19.7	9.5	2.1	32.7
Age Distribution of Positive Males (%)	8.1	14.1	65.7	3.2	6.9	1.7	0.2	100%
**FEMALES****	**< 2Yrs**	**2-4 yrs**	**5-18 yrs**	**19-24 yrs**	**25-49yrs**	**50-64yrs**	**≥ 65 yrs**	**Total**
Number positive	95	182	725	62	225	48	7	1344
Number tested	659	567	1717	317	1159	401	185	5005
Positivity rate (%)	14.4	32	42.2	19.5	19.4	11.9	3.8	26.8
Age Distribution of Positive Females (%)	7.0	13.6	54.0	4.6	16.7	3.6	0.5	100%
Ratio of M/F tested	1.2	1.13	0.99	0.35	0.41	0.60	0.77	0.82

Table S 1. *Cont.*

**Total positive patients**	205	372	1612	105	318	71	10	2693*
Total tested patients	1472	1209	3419	427	1633	641	327	9128*
Positive Rate (%)	13.9	30.7	47	24.5	19.4	11.0	3.1	29.5
Age Distribution of Positive patients (%)	7.6	13.8	59.9	3.9	11.8	2.6	0.4	100%
Ratio of M/F tested	1.2	1.13	0.99	0.35	0.41	0.60	0.77	0.82
Estimated infected MC-symptomatic	3092	5614	24367	1586	4800	1058	163	40679
Estimated infected MC-asymptomatic	4393	7978	34626	2255	6821	1503	231	57807
Total infected MC	7485	13592	58993	3841	11621	2561	394	98486
MC populations		74434**	190198	78234	328640	171399	110423	953328
Percentage MC infected-1st Wave		28.3	31.0	4.9	3.5	1.5	0.4	10.3

48/2741 positive and 48/9176 tested patients did not have gender available and were excluded from this analysis. ** Milwaukee county population < 5 years of age.

Table S 2. Infection rates per 100,000 MC population by age and gender.

	**Males**	**Females**	**Total**
**< 5**	1109.0	1067.6	1101.9
**5-18 yrs**	1197.7	1015.1	1131.4
**19-24**	187.6	271.9	231.8
**25-49**	81.6	189.3	138.5
**50-64**	39.2	75.7	59.0
**>=65**	9.7	14.7	12.7
**5-24 yrs**	958.7	835.2	916.2

Table S 3. Hospitalization rates per 100,000 MC population by age and gender.

	**Males**	**Females**	**Total**
**< 5**	60.3	37.2	49
**5-18 yrs**	19	17.7	18.4
**19-24**	6.5	19.5	13
**25-49**	6.4	5	5.7
**50-64**	6.3	5.8	6.1
**>=65**	0	4.7	2.8
**5-24 yrs**	16.1	18.1	17.1

Figure S 1. Influenza A virus activity over the 2008-2009 winter spring and summer seasons by subtypes detected in Milwaukee, WI. H1N1h and H3N2 are the seasonal human subtypes of influenza A. H1N1pdm is the pandemic H1N1 subtype. Arrow 1: surveillance criteria changed to testing only high risk or hospitalized patients Arrow 2: Schools closed in Milwaukee

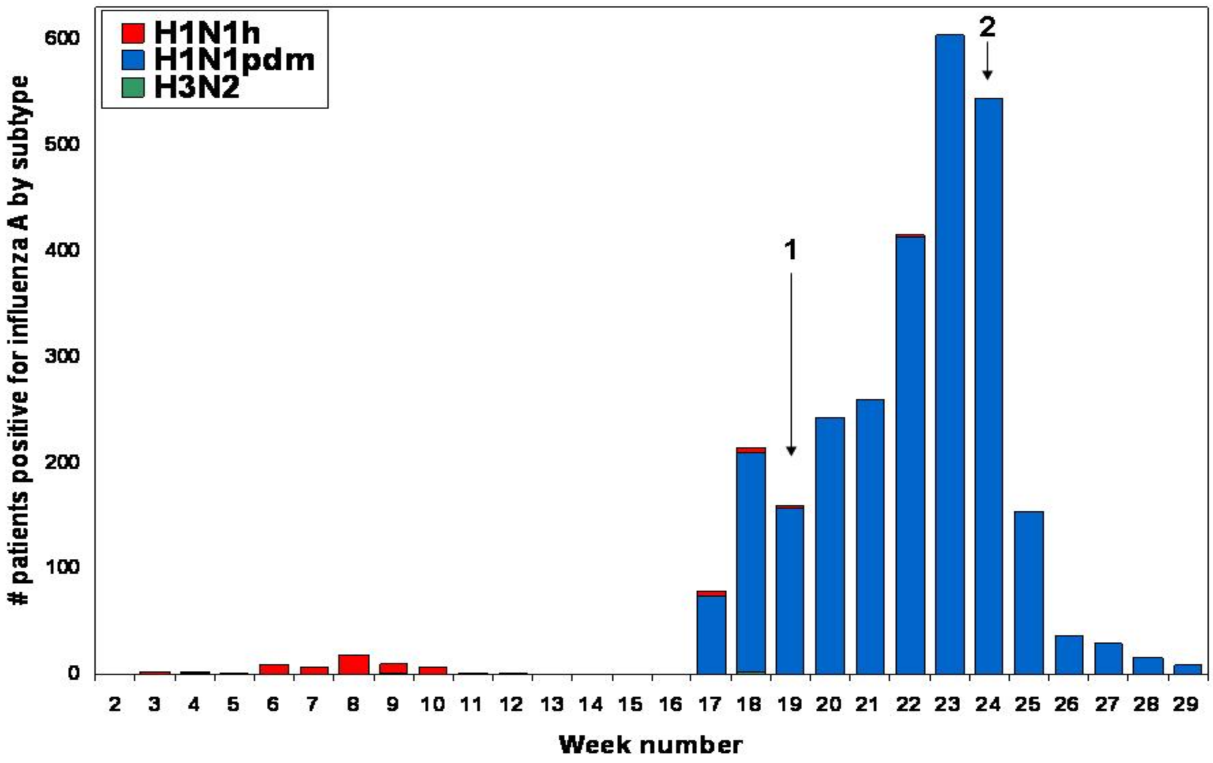

Figure S 2. Age distribution of patients infected with the H1N1pdm virus, by week of enhanced surveillance; * Week of school closure.

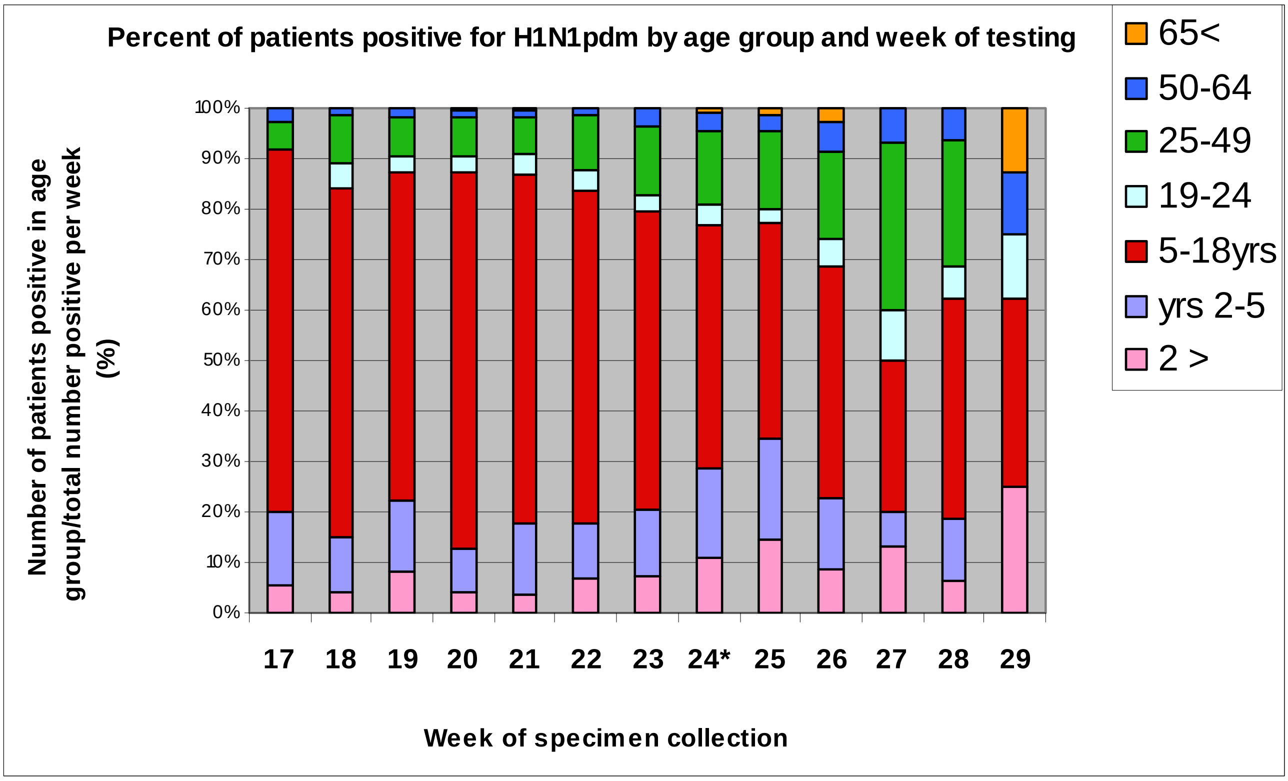

Figure S 3. Age distribution of patients infected with the H1N1pdm virus, by week of enhanced surveillance.

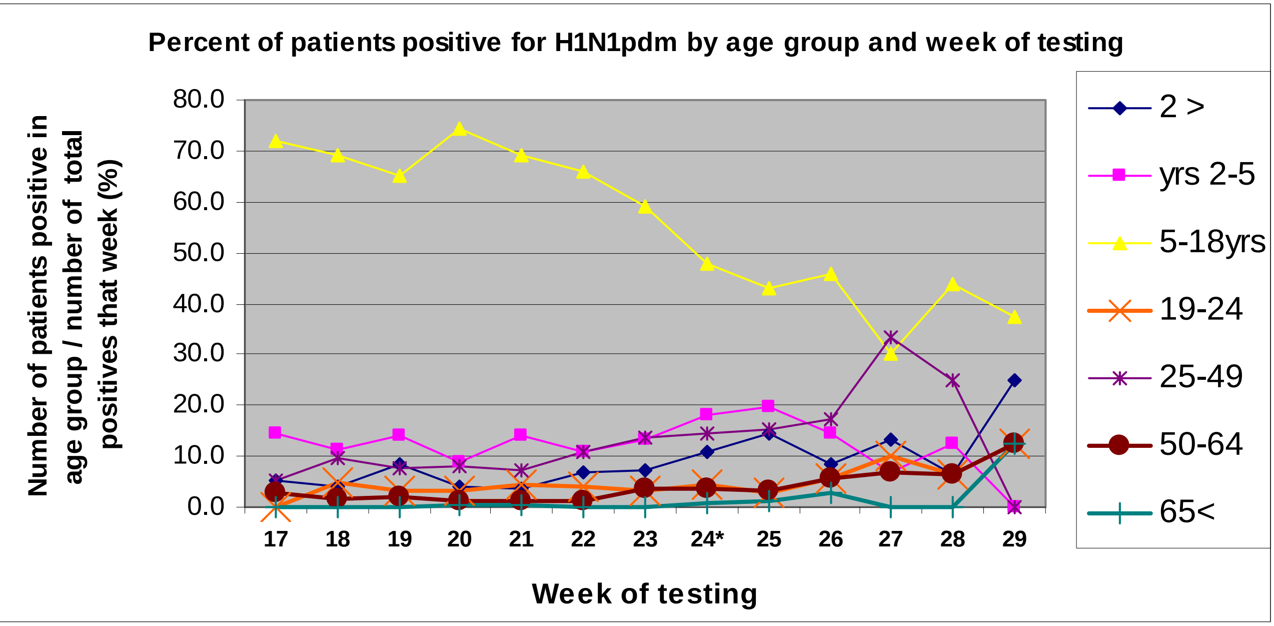

* Week of school closure.

Figure S 4. Age distribution of H1N1pdm laboratory detection rates, by week of enhanced surveillance.

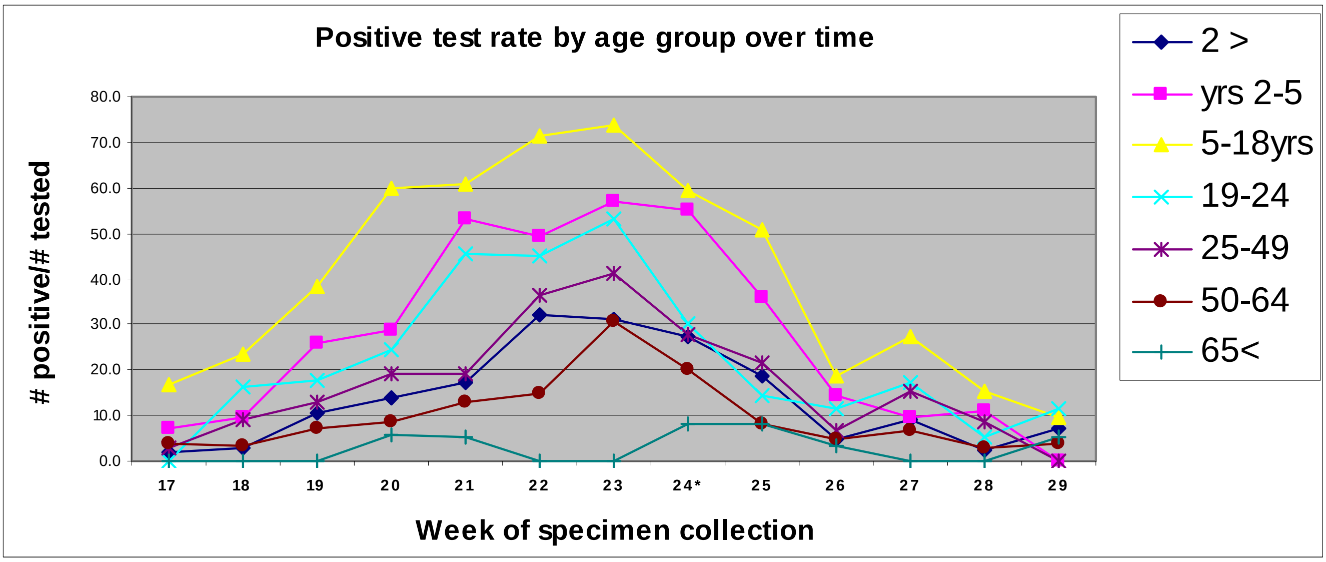

* Week of school closure.

Figure S 5. H1N1pdm Infection Rates per 100,000 MC population by age and sex.

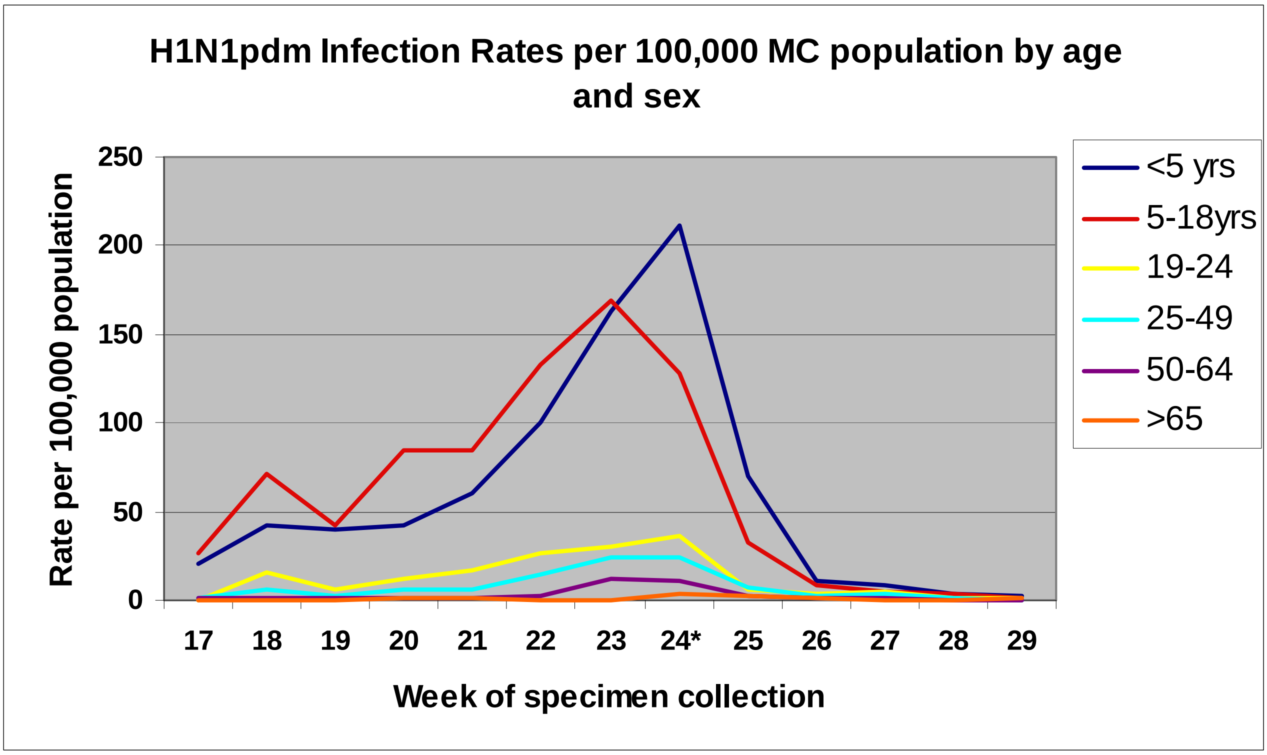

* Week of school closure.

Statistical methods for estimating number of infected individuals in the first pandemic wave.

The ideal way to allow for disparity in demographics of the sample and the Milwaukee population would be to weight differently depending on age, gender and race over the weeks since we know that there was variation in all of these. However the small numbers (N=614) enrolled in targeted surveillance precluded weightage using all three demographic variables. Data from the American community survey (1) was examined to see how the age distribution and gender distribution varied over races. Although there was some variation it was relatively low with the major disparity being the racial distribution. Therefore we weighted our estimates taking into account the Milwaukee rates for race.

In the calculation we only weighted the AA, Caucasian and Hispanic races since our other races were extremely low in numbers. Since ILI data for MC for the last 4 weeks (week 26-29 data not published) and the "symptomatic" data for the first 3 weeks (targeted surveillance initiated in week 20) were not available, the data were assumed to be approximately symmetrical and values form the other side of the curve were imputed. Because of the variation of week by week which could be artificial, we chose to also estimate the variation over time by using a moving average of three weeks since a subject could be positive over two weeks and possibly lasting up to three weeks, at the end of the period we assumed 0 cases.

Table S 4.: Adjusting positive test rates in symptomatic subjects in targeted surveillance.

Row #	Race	# Tested (A)*	# Positive (B)*	Targeted surveillance Population Proportion (C)*	MC Population Proportion (D)*	Relative weight (E)*	Absolute weight (F)*	Adjusted # Tested (G)*	Adjusted # Positive (H)*	Overall weight (I)*
**1**	Latino	82		0.33	0.12	0.36	0.12	29.48		
**2**	Latino		23						8.27	
**3**	Blacks	92		0.37	0.25	0.67	0.22	54.74		
**4**	Blacks		16						9.52	
**5**	Caucasians	72		0.29	0.58	1.97	0.66	161.73		
**6**	Caucasians		3						6.74	
**7**	**Total**	246	42			3	1		24.53	0.58

*E=D/C; F1=E1/E7; G=F*A7; H2= B2/A1; I=H7/B7

Table S 5. Adjusting positive test rates in asymptomatic subjects in active surveillance.

Row #	Race	# Tested (A)*	# Positive (B)*	Targeted surveillance population Proportion (C)*	MC Population Proportion (D)*	Relative weight (E)*	Absolute weight (F)*	Adjusted # Tested (G)*	Adjusted # Positive (H)*	Overall weight (I)*
**1**	Latino	70		0.2	0.12	0.6	0.22	77.8		
**2**	Latino		1						1.106	
**3**	Blacks	156		0.44	0.25	0.56	0.2	72		
**4**	Blacks		4						1.857	
**5**	Caucasians	128		0.36	0.58	1.6	0.58	204		
**6**	Caucasians		0							
**7**	**Total**	354	5			2.76	1	353.8	2.96	0.59

*E=D/C; F1=E1/E7; G=F*A7; H2= B2/A1; I=H7/B7.

Table S 6. Calculation of number of infected symptomatics in MC by week

week #	%ILI[@] [A]	% SS* who have ILI (active surveillance) [B]	% SS in the community [C]	% of SS who were positive in active surveillance [D]	MC Population [E]	Number of SS [F]	Number of infected SS [G]	Number of infected SS (adjusted) [H]	Weight applied [I]
17	5.00	25.00	20.00	8.30	953328	190666	15825	9242	0.58
18	2.50	37.50	6.67	7.70	953328	63555	4894	2858	0.58
19	0.80	37.50	2.13	5.00	953328	20338	1017	594	0.58
20	1.50	50.00	3.00	18.75	953328	28600	5362	3132	0.58
21	0.20	28.00	0.71	12.00	953328	6809	817	477	0.58
22	2.00	53.85	3.71	15.38	953328	35409	5448	3181	0.58
23	2.40	60.87	3.94	45.65	953328	37588	17160	10021	0.58
24	4.00	65.79	6.08	18.42	953328	57962	10677	6236	0.58
25	2.20	41.46	5.31	5.00	953328	50582	2529	1477	0.58
26	1.80	53.85	3.34	7.69	953328	31868	2451	1432	0.58
27	0.20	37.50	0.53	8.33	953328	5084	424	247	0.58
28	1.30	37.50	3.47	0.00	953328	33049	0	0	0.58
29	0.60	25.00	2.40	13.33	953328	22880	3051	1782	0.58
								40679	

C=(A/B) X100; F= E X C; G= D X F; H=G X I

* SS, symptomatic subjects, [@,] ILI, influenza like illness
